# The association between hypodontia and dental development

**DOI:** 10.1007/s00784-015-1622-1

**Published:** 2015-10-13

**Authors:** Brunilda Dhamo, Strahinja Vucic, Mette A. R. Kuijpers, Vincent W. V. Jaddoe, Albert Hofman, Eppo B. Wolvius, Edwin M. Ongkosuwito

**Affiliations:** Department of Oral & Maxillofacial Surgery, Special Dental Care and Orthodontics, Erasmus University Medical Centre, PO Box 2040, 3000 CA Rotterdam, The Netherlands; The Generation R Study Group, Erasmus University Medical Centre, Rotterdam, The Netherlands; Department of Orthodontics and Craniofacial Biology, Radboud University Nijmegen Medical Center, Nijmegen, The Netherlands; Department of Epidemiology, Erasmus University Medical Centre, Rotterdam, The Netherlands

**Keywords:** Tooth agenesis, Teeth development, Dental age, Dutch dental age standards

## Abstract

**Objectives:**

In this cross-sectional study, we aimed to investigate the pattern of hypodontia in the Dutch population and determine the association between hypodontia and dental development in children with and without hypodontia, applying three different standards, Dutch, French Canadian, and Belgian, to estimate dental age.

**Methods:**

We used dental panoramic radiographs (DPRs) of 1488 children (773 boys and 715 girls), with a mean age of 9.76 years (SD = 0.24) participating in a population-based cohort study in Rotterdam, the Netherlands, born in 2002–2004, and 452 children (219 boys and 233 girls) with a mean age of 9.83 years (SD = 1.09) participating in a mixed-longitudinal, interdisciplinary population-based cohort study in Nijmegen, the Netherlands born in 1960–1968.

**Results:**

The prevalence of hypodontia in the Generation R Study was 5.6 % (*N* = 84) and 5.1 % (*N* = 23) in the Nijmegen Growth Study. Linear regression analysis showed that children with hypodontia had a 0.37 [95 % CI (−0.53,-0.21)] to 0.52 [95 % CI (−0.76,-0.38)] years lower dental age than children without hypodontia. The ordinal regression analysis showed a delay in development of mandibular second premolars [1.68 years; 95 %CI (−1.90,-1.46)], mandibular first premolars [0.57 years; 95 % CI (−0.94,-0.20)], and mandibular second molars [0.47 years; 95 % CI (−0.84,-0.11)].

**Conclusion:**

These findings suggest that children with hypodontia have a delayed dental development.

**Clinical relevance:**

The delay of dental development in children with hypodontia should be taken into consideration and therefore orthodontists should recognize that a later start of treatment in these patients may be necessary.

**Electronic supplementary material:**

The online version of this article (doi:10.1007/s00784-015-1622-1) contains supplementary material, which is available to authorized users.

## Introduction

Hypodontia is defined as the developmental absence of one or more primary or secondary teeth, excluding the third molars [1, 2]. It is classified according to the number of absent teeth: mild if one tooth is absent, moderate if two to five teeth are absent, and severe if more than six teeth are absent [3, 4]. It is the most recognized congenital dental anomaly, and therefore presents a frequent clinical problem encountered by orthodontists and other dental professionals [5–7].

Most studies in which the prevalence of hypodontia was investigated were performed in Caucasians. These studies showed a prevalence of hypodontia of 5.5 % in European, 3.9 % in North American, and 6.4 % in the Australian population [8]. The highest prevalence of hypodontia, 6.9 %, was found in an Asian population [9]. Investigations in other populations are scarce. In the Dutch population, the prevalence of hypodontia is similar to the prevalence observed in European studies and is estimated to be 5 % [10]. The prevalence of hypodontia is substantially higher in some disorders such as ectodermal dysplasia [11, 12], Down syndrome [13, 14], Witkop syndrome [15, 16], and cleft lip or palate [17]. The most frequently affected tooth is the mandibular second premolar, followed by the maxillary second incisor and the maxillary second premolar [8]. Although statistically significant differences were inconsistent throughout the literature, most reported a higher occurrence of hypodontia in females [18–20].

Few studies have investigated whether an association exists between non-syndromic hypodontia and dental development [21–24]. In a previous study, a significantly delayed dental development in subjects with hypodontia was reported [22]. Furthermore, the same authors reported that isolated hypodontia can impact the development of adjacent teeth by decreasing crown size, changing crown and root morphology, delaying development, or inducing taurodontism. Another report identified a similar result of delayed dental development in children with hypodontia [21]. On the other hand, researchers reported a non-significant difference of dental development between children with hypodontia and their matched controls [24]. These inconsistent findings prompted us to conduct a study with a large-sized sample in the general population.

In this cross-sectional study, we aimed to determine the association between hypodontia and dental development in children with and without hypodontia using three different standards, Dutch, French Canadian, and Belgian, to obtain the best estimation of dental age in relation to chronological age.

## Materials and methods

### Study population

Our cross-sectional study aims to represent Dutch population over time so we used 1940 dental panoramic radiographs (DPR) of 1940 children, obtained from two cohorts in different cities in the Netherlands, the Generation R Study in Rotterdam and the Nijmegen Growth Study (Table 1).

**Table 1:** Characteristics of children included in the study (*N* = 1940)

	Generation R sample (*N* = 1488)			Nijmegen sample (*N* = 452)		
	Controls (*N* = 1404)	Hypodontia (*N* = 84)	*P* value*	Controls (*N* = 429)	Hypodontia (*N* = 23)	*P* value*
Gender (*N*, %)			0.94			0.62
Boys	729 (52)	44 (52)		209 (52)	10 (52)	
Girls	675 (48)	40 (48)		220 (48)	13 (48)	
Age (years; mean, SD)	9.76 (0.24)	9.73 (0.20)	0.30	9.85 (1.05)	9.47 (1.56)	0.10
Ethnicity (*N*, %)			0.24			
Dutch	934 (67)	52 (62)		429 (100)	23 (100)	
Non-Dutch	438 (31)	32 (38)		0	0	
Maternal age (years; mean, SD)	30.82 (4.89)	31.34 (5.14)	0.35	29.86 (5.79)	30.92 (5.56)	0.46
Dental age (years; mean, SD)						
Dutch standards						
Method 1^a^	10.40 (0.78)	10.03 (0.75)	<0.05	10.60 (1.40)	9.86 (1.68)	<0.05
Method 2^b^	10.40 (0.78)	9.90 (0.88)	<0.05	10.60 (1.40)	9.81 (1.65)	<0.05
French-Canadian standards						
Method 1^a^	11.31 (1.15)	10.76 (1.07)	<0.05	11.57 (1.61)	10.86 (1.94)	<0.05
Method 2^b^	11.32 (1.12)	10.62 (1.18)	<0.05	11.61 (1.63)	10.77 (1.86)	<0.05
Belgian standards						
Method 1^a^	13.56 (2.95)	13.11 (2.80)	0.17	14.22 (3.41)	13.73 (3.71)	0.50
Method 2^b^	13.57 (2.95)	13.01 (2.77)	0.09	14.22 (3.41)	13.63 (3.62)	0.42

*N* number of children, *SD* standard deviation

*Differences were tested using independent *t* test for continuous variables and chi-squared test for categorical variables

Dental age was calculated if both matching mandibular teeth were missing by scoring them: ^a^as a developmental stage calculated from regression equations developed by [30], ^b^as a developmental stage of the (left) matching maxillary tooth

The Generation R Study is a population-based prospective cohort study from fetal life until young adulthood established in the city of Rotterdam in the Netherlands [25–27]. From the still ongoing fourth examination phase, we used 1488 DPRs taken of 773 girls and 715 boys, with a mean age of 9.76 ± 0.24 years and born between 2002 and 2003. At the start of each phase, mothers and their partners received written and oral information about the study and they were asked for their written informed consent. The study was approved by the Medical Ethics Committee of the Erasmus Medical Centre in Rotterdam, the Netherlands (MEC-2012-165).

The second sample was derived from the Nijmegen Growth Study, a mixed-longitudinal, interdisciplinary population-based cohort study in healthy Dutch children conducted from 1971 to 1976 at the Radboud University Medical Centre in Nijmegen, the Netherlands. The design of this cohort was described in the past [28]. Children were enrolled at 4, 7, and 9 years of age and followed until 9, 12, and 14 years. From this cohort, we used 452 DPRs of 219 boys and 233 girls, with a mean age of 9.83 ± 1.09 years and born between 1960 and 1968. Prior to the collection of general, physiological, dental, and anthropometric measurements of children, informed consents were obtained from their parents. Children who were not born in the Netherlands and nonwhite children were excluded from the study. The participants in this study had no recognizable syndrome associated with hypodontia.

### The assessment of hypodontia

One experienced examiner ascertained hypodontia from the DPRs. Children were included in the hypodontic group if they missed at least one tooth (no sign of formation or calcification showed in DPR).

### Dental development assessment

Dental development was defined using the Demirjian method [29]. One experienced examiner determined one of the eight developmental stages (A, B, C, D, E, F, G, and H) for each of the seven teeth located in the lower left quadrant. In order to estimate the developmental stage of the hypodontic teeth, we applied two methods. In Method 1, we applied regression equations [30], which take into account the development of the remaining teeth in the lower left quadrant and age of a child to calculate dental age. In Method 2, we assessed the stage of development for a hypodontic tooth in the left mandible from the corresponding right mandibular tooth if it was present or from a corresponding maxillary tooth if that tooth was missing in both sides of the mandible. In the case when no corresponding tooth was present, stage 0 was assigned to that tooth. Obtained stages of dental development were used to calculate the dental maturity score by summing up the weighted scores from Dutch, French-Canadian, and Belgian dental age standards [29, 31, 32]. Lastly, we used standard tables to convert the dental maturity score to dental age [29, 31, 32].

### Statistical analysis

We calculated the intra-class correlation coefficient to determine agreement between two independent examiners who assessed the presence of hypodontia and stages of development (A to H) for each of the seven left mandibular teeth in a subsample of 20 DPRs from the study population.

The association between hypodontia and dental development in children was analyzed with linear regression models and by adjusting for confounders in three consecutive steps. In the first model, we analyzed the crude dependence of dental age on the hypodontia status of children. In the second model, we additionally adjusted for gender, age, and study population. Study population was taken into account to avoid any possible cohort effect. Lastly, in the third model, variables ethnicity and maternal age at the birth of a child were added. Maternal age at birth was added because previous studies showed that certain maternal factors may have an influence on the condition of hypodontia and dental development of children [33].

To study the association between hypodontia and the developmental stage for each of the observed teeth separately from the lower left quadrant, we performed an ordinal regression analysis. Dental development stages (A to H) were converted into numbers (1 to 8) and used as a dependent variable while the independent variables were added in three consecutive steps, as previously described for the linear regression analysis. In order to avoid possible errors of the two methods for assigning the stage of development 0 to hypodontic teeth, we excluded stage 0 from being a dependent variable in the ordinal regression model.

We tested for interaction terms between gender, ethnicity, and hypodontia in relation to dental development. Since no significant interaction terms were found, we did not stratify our analyses for these interaction terms. The Markov Chain Monte Carlo imputation method was used to reduce potential bias associated with missing data on maternal age at birth in 99 children (5 %) [34]. As a result, five imputed datasets were generated from which a pooled effect estimate was calculated. The result was considered statistically significant for a *P* value ≤0.05. All statistical analyses in this study were performed using statistical software SPSS version 21.0 (SPSS Inc. Chicago, IL, USA).

## Results

### Inter-examiner agreement for the study population

The inter-examiner reliability of the study population was performed by two independent researchers in a subsample of 20 DPRs. We found an excellent agreement between the examiners for the scoring of the central incisors, with an intra-class correlation coefficient (ICC) equal to 1.00. The intra-class correlation coefficient was the lowest for the first molars (ICC = 0.49), while the range of ICC values for the rest of the scored teeth ranged from good to excellent (ICC = 0.79–0.94).

### Prevalence of hypodontia

The distribution of tooth agenesis is presented in Supplementary Table S1. The prevalence of hypodontia in the Generation R Study was 5.6 % (*N* = 84) and 5.1 % (*N* = 23) in the Nijmegen Growth Study. The most common hypodontic teeth in the Generation R Study and the Nijmegen Growth Study were the mandibular second premolars, 51.8 % (*N* = 72); 50.0 % (*N* = 20), respectively; *P* = 0.84, and the maxillary lateral incisor, 15.8 % (*N* = 22); 27.5 % (*N* = 11), respectively; *P* = 0.09. None of the children had more than five hypodontic teeth. The prevalence of hypodontia was similar in both sexes in the Generation R Study sample (*P* = 0.94) and the Nijmegen Growth study sample (*P* = 0.62) (Table 1).

### Crude analysis

**Fig. 1:**
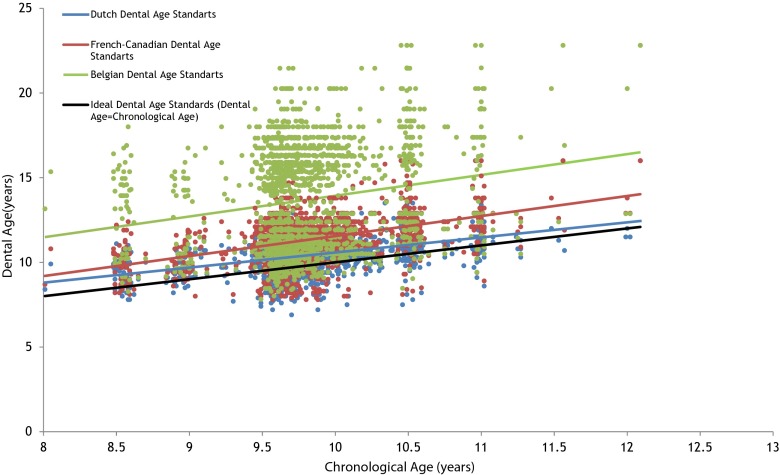
Dental age of study population assessed from Dutch, French-Canadian, and Belgian standards are presented as a function of chronological age of children

The calculated dental age using Dutch (10.35 ± 0.91), French-Canadian (11.29 ± 1.35), and Belgian (13.65 ± 3.07) standards was statistically significantly higher, than the chronological age (9.78 ± 0.57) of children (*P* ≤ 0.05) (Table 1). We observed a statistically significant lower dental age in children with hypodontia, compared to controls by applying the two methods to score hypodontic teeth using Dutch standards, French-Canadian standards, and Belgian standards (*P* ≤ 0.05). The mean difference between chronological and dental age was the least when using Dutch standards. For this reason, dental age defined by Dutch standards was used in the linear regression analysis (Fig. 1).

### Linear regression analysis: association between hypodontia and dental age

The association between dental age and hypodontia was investigated by three linear regression models separately for each of the two methods and is presented in Table 2. Univariate linear regression analysis showed that a child with hypodontia had a 0.46 [95 % CI (−0.65,-0.27)] to 0.57 [95 % CI (−0.76,-0.38)] years lower dental age compared to a child without hypodontia. After additionally adjusting Model 2 for age, sex, and study population, the effect estimate of the hypodontia status variable changed, resulting in a 0.36 [95 % CI (−0.52,-0.20)] to 0.52 [95 % CI (−0.68,-0.35)] years lower dental age in children with hypodontia. The effect estimates and statistical significance barely changed by taking into account the ethnicity of a child and maternal age at birth, in the fully adjusted model.

**Table 2:** Linear regression models: association between hypodontia and dental age using Dutch standards

	Model 1			Model 2			Model 3		
	β	95 % CI	*P* value	β	95 % CI	*P* value	β	95 % CI	*P* value
Method 1^a^									
Hypodontia									
No (ref.)	0	–	–	0	–	–	0	–	–
Yes	−0.46	(−0.65,-0.27)	<0.05	−0.36	(−0.52,-0.20)	<0.05	−0.37	(−0.53,-0.21)	<0.05
Method 2^b^									
Hypodontia									
No (ref.)	0	–	–	0	–	–	0	–	–
Yes	−0.57	(−0.76,-0.38)	<0.05	−0.52	(−0.68,-0.35)	<0.05	−0.52	(−0.69,-0.36)	<0.05

Model 1 is the crude dependence of dental age on the hypodontia; Model 2 was additionally adjusted for age, gender, and study population; and Model 3 was adjusted for variables used in previous model and additionally for ethnicity and maternal age at birth of a child

*β* regression coefficients, *CI* confidence interval, *ref.* reference

Dental age was calculated if both matching mandibular teeth were missing by scoring them: ^a^as a developmental stage calculated from regression equations developed by [30]; ^b^as a developmental stage of the (left) matching maxillary tooth

### Ordinal regression analysis: association between hypodontia and stages of dental development

**Fig. 2:**
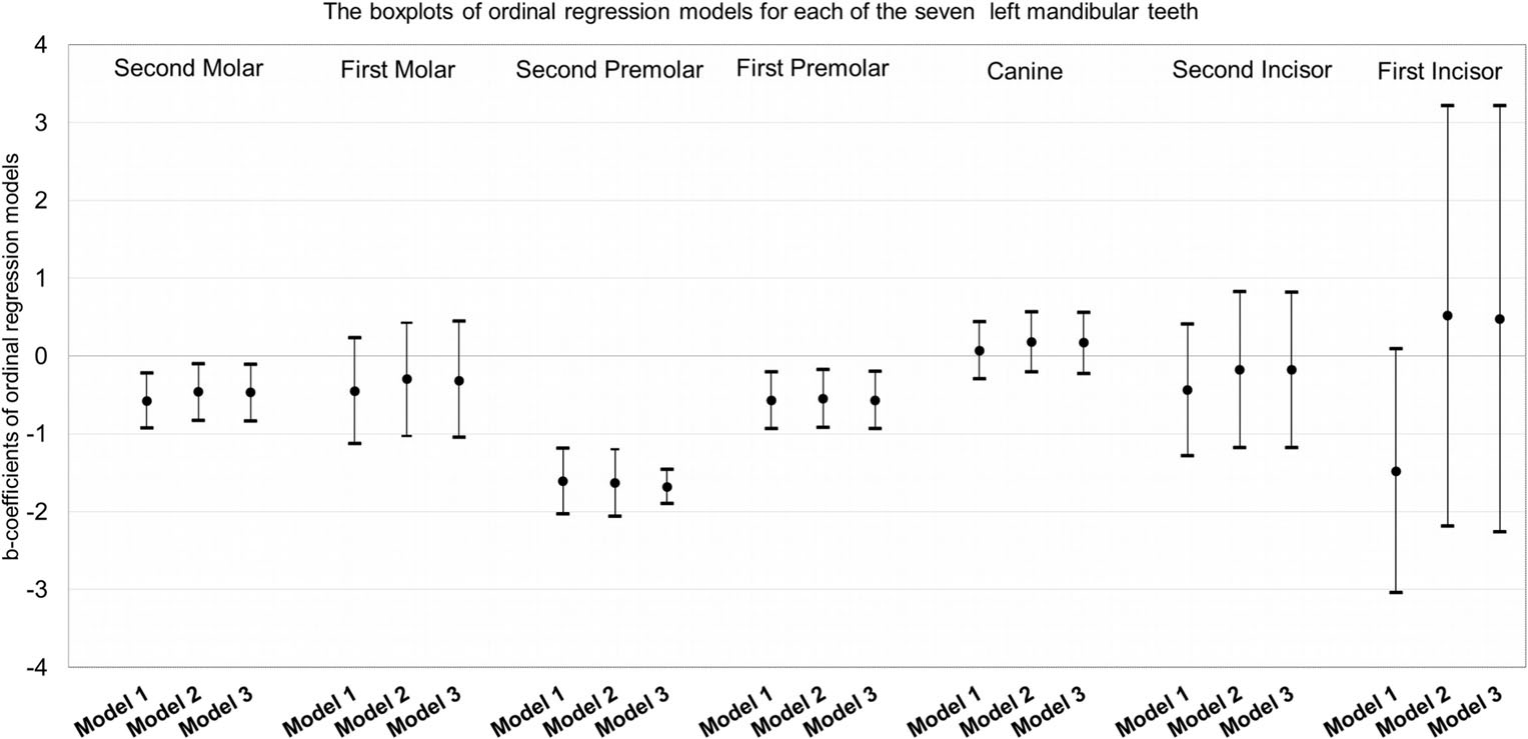
Association of hypodontia with stages of dental development for each of the seven left mandibular teeth, expressed by estimates of b-coefficients and 95 % confidence intervals; assessed from ordinal regression model using developmental stage (A/1, B/2, C/3, D/4, E/5, F/6, G/7, H/8) as a dependent variable and hypodontia status (No-ref., Yes) as a determinant in Model 1. Model 2 was additionally adjusted for age, gender, and study population. Model 3 was adjusted for variables used in previous model and additionally for ethnicity and maternal age at birth of a child

Results for the left mandibular second molar, first molar, second premolar, first premolar, canine, and lateral and central incisors are shown in Fig. 2. The following regression coefficients and *P* values are reported from the third model (fully adjusted model) of ordinal regression. The greatest difference in obtained developmental stages was observed for the left mandibular second premolar, where the results of the ordinal regression analysis showed that children with hypodontia tend to have lower dental developmental stages than the controls [−1.68 years; 95 % CI (−1.90,-1.46)]. In addition, similar negative and significant associations were observed for the left mandibular first premolar [−0.57 years; 95 % CI (−0.94,-0.20)] and for the left mandibular second molar [−0.47 years; 95 % CI (−0.84,-0.11)]. Developmental stages between children with hypodontia and controls did not significantly differ for the central incisor [0.48 years; 95 % CI (−2.26, 3.22)], lateral incisor [−0.18 years; 95 % CI (−1.18, 0.82)], canine [0.17 years; 95 % CI (−0.23, 0.56)], and first molar [−0.32 years; 95 % CI (−1.05, 0.42)].

## Discussion

**Fig. 3:**
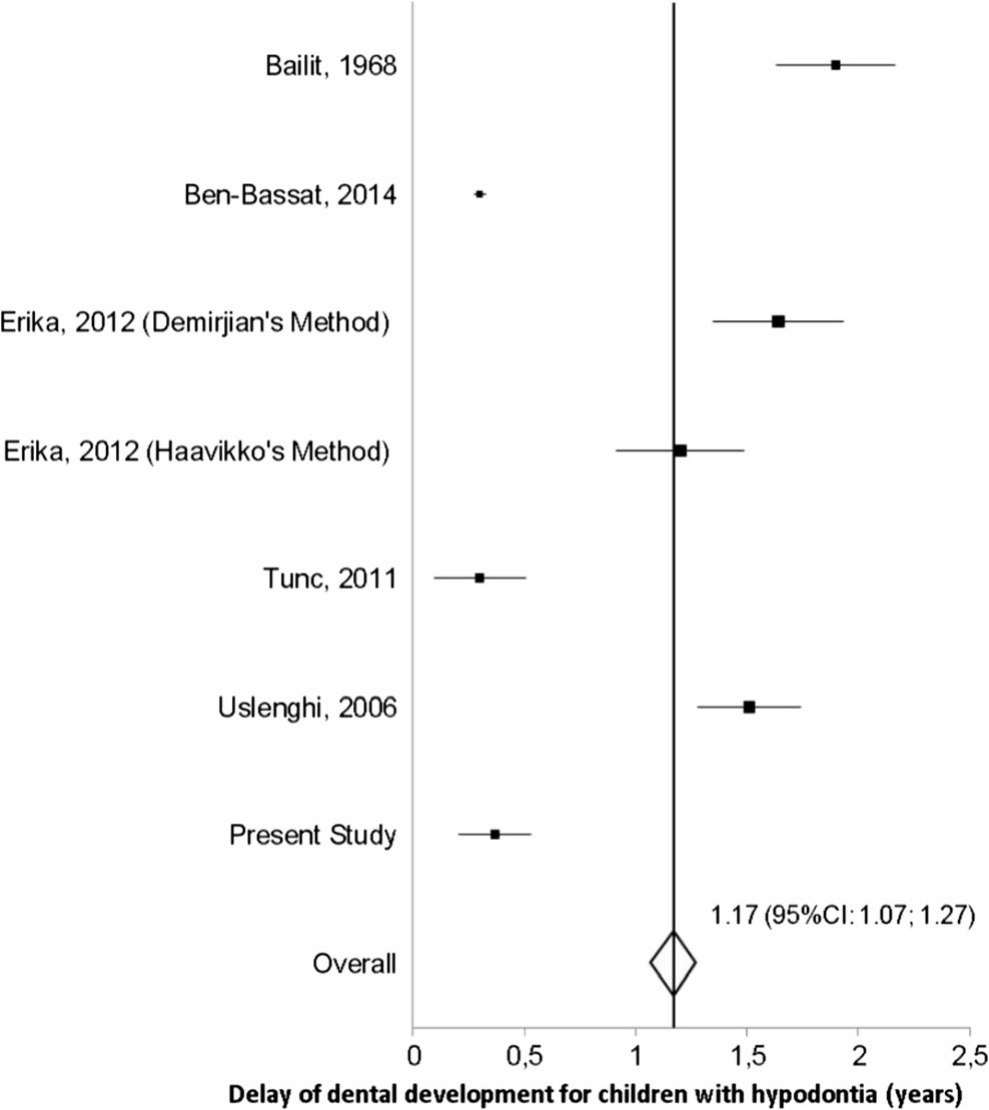
The forest plot of studies on the association between hypodontia and dental development

The findings of our study suggest a significant delay of 0.37–0.52 years in dental development of children with hypodontia, supporting the overall mean of earlier studies of 1.04 years delay in dental development presented in Fig. 3. Different results on the association between hypodontia and dental development have been observed possibly because of different methods used to define the developmental stage of hypodontic teeth (Table S2). Accordingly, previous investigators have proposed different techniques to tackle this problem. Uslenghi (2006) used a method and data from Haavikko’s scoring system to overcome the problem of scoring a hypodontic tooth [35]. On the other hand, Tunc [36] used an adapted Demirjian method which relies on the development stages of three teeth only: left mandibular canine, first premolar, and second molar. We used two methods to estimate the developmental stage of the hypodontic teeth. The advantage of using Method 1 in patients with hypodontia is that the developmental stage is obtained from mathematical formulas for each missing tooth separately [30]. By using Method 2, we tested the suitability of regression equations from Method 1 as they were derived from a Finnish population. Method 2 may be more suitable when assessing dental age in children with mild hypodontia because in using Method 1, the underlying population stays an important factor in establishing the imputations formulas. However, in cases of severe hypodontia in which the same tooth is missing in all four quadrants, Method 1 may be more advantageous for the calculation of dental age than Method 2. The limitation of the two methods used in this study might be the dependence of calculated dental age on the estimated stage of development for the hypodontic tooth. We tried to overcome the problem related to assessing dental development in children with hypodontia by using ordinal regression models in which stage 0 of development of every left mandibular tooth (hypodontic teeth) was not used in the analysis, and the effect of hypodontia is assessed directly from the eight stages of dental development for every single tooth.

A combination of several methods for determining dental development is generally recommended for a better estimation of dental age [24]. We used three different dental age standards (Dutch, French-Canadian, and Belgian) in order to approach dental age to chronological age of the children the best. The French-Canadian standard is the most used in literature although studies were not performed in Canada. Our assumptions were that dental age assessed by Dutch standards would resemble chronological age of our sample better than Belgian Standards and that dental age assessed by Belgian standards would resemble chronological age better than French-Canadian, because of the geographical proximity of the Dutch and Belgian population. Belgian standards were indeed better than French-Canadian’s in defining dental age for boys but the estimated dental age for girls was at least 6 years higher than their real age. The calculations we did showed that the inaccuracy of Belgian standards was not in the scores they presented, but in the polynomial equations that they used to define dental age for girls. Although chronological age was closer to dental age estimated from Dutch standards than to dental age estimated from French-Canadian or Belgian standards, still a statistically significant difference existed between Dutch dental age and chronological age. A better approach of Dutch standards needs to be performed in a larger sample of Dutch population in the future.

The frequency of hypodontia in the cohorts of the Nijmegen Growth Study and the Generation R Study coincided with an earlier prediction of 5 % in the Dutch population [10]. It has been hypothesized that prevalence of hypodontia in permanent teeth increases over the years [37]. We compared the prevalence of hypodontia in 1970 and 2010 between the cohorts of the Nijmegen Growth Study (5.1 %) and the Generation R Study (5.6 %) and found no statistically significant difference. A higher prevalence has been reported in females than in males, with a ratio of 3:2 [8] but in our study the frequency of hypodontia did not differ by gender or by ethnicity.

The results from ordinal regression models showed that the delay in dental development was caused mainly by the second premolar [1.68 years; 95 % CI (−1.90,-1.46)], the last in the row of premolars which is also the most prevalent hypodontic tooth in our study, consistently with previously published literature [8, 9]. As a consequence of evolution, what is less needed is going to disappear naturally [38]. This may explain the major absence of the third molar, which is the latest developing tooth and molar, and may be explained in the same way for the last premolar, the second premolar and lateral incisor [39]. At the age of ten, we observed little variation for central, lateral incisors, and first molars because they were in the final stage of development, common for 9–10-year-old children. However, to test whether there is delayed dental development of incisors and first molars, DPRs of children of younger ages need to be taken when these teeth have not yet reached the final stage of development. The effect of hypodontia in the development of the canine, important in our dentition, was not statistically significant. Cases of hypodontic canines are rarely reported [8, 9]. Following this line of thought, the trend of tooth loss throughout the evolution of mankind could explain the association between hypodontia and delayed dental development. Although an association between delayed dental development and hypodontia was found in our cross-sectional study, it currently remains uncertain whether hypodontia causes delay of dental development or vice versa [40]. The nature of this association would be better determined by genetic investigations in humans, taking into consideration the different pathways of *PAX9*, *MSX1*, and *AXIN2* acting on both hypodontia and delayed dental development [4, 41, 42].

## Conclusions

The findings of our study indicate a lower dental age in children with hypodontia. The delay varied from 0.37 to 0.52 years of dental age between the groups of hypodontia and non-hypodontia and the difference in development was mostly pronounced for the second lower premolars, first lower premolar, and second lower molars.

## Electronic supplementary material

Below is the link to the electronic supplementary material.

ESM 1 (DOCX 23 kb)
