# Supplementary material for: The association between hypodontia and dental development
Source: Clin Oral Investig. 2015 Oct 13;20:1347–54. doi: 10.1007/s00784-015-1622-1 (PMC4914514; doi:10.1007/s00784-015-1622-1)
Supplement: Supplementary file 1 — (DOCX 23 kb) [file 784_2015_1622_MOESM1_ESM.docx]

**Supplementary material:**

**THE ASSOCIATION BETWEEN HYPODONTIA AND DENTAL DEVELOPMENT**

Brunilda Dhamo^1,2^, Strahinja Vucic^1,2^, Mette AR Kuijpers^3^, Vincent WV Jaddoe^2,3^, Albert Hofman^2,3^, Eppo B Wolvius^1,2^, Edwin M Ongkosuwito^1,2,*^

^1^Department of Oral & Maxillofacial Surgery, Special Dental Care and Orthodontics, ^2^The Generation R Study Group, Erasmus University Medical Centre, The Netherlands, ^3^Department of Epidemiology, Erasmus University Medical Centre, The Netherlands, ^4^Department of Orthodontics and Craniofacial Biology, Radboud University Nijmegen Medical Centre, The Netherlands

*Corresponding author: Edwin M Ongkosuwito

Department of Oral & Maxillofacial Surgery, Special Dental Care and Orthodontics,

Erasmus University Medical Centre

PO Box 2040, 3000 CA Rotterdam, The Netherlands

Tel + 31 10 7036426

Email: e.ongkosuwito@erasmusmc.nl

**Supplementary tables and figures**:

Table S1: The distribution of hypodontic teeth in the sample of the Generation R Study and the Nijmegen Growth Study.

Table S2: General summary of studies performed on the association between hypodontia and dental development.

| **Table S1. Distribution of hypodontic teeth in children** | | | | | | |
| --- | --- | --- | --- | --- | --- | --- |
|  | Generation R | | | Nijmegen | | |
| FDI tooth code | Boys | Girls | Total | Boys | Girls | Total |
| 11 | - | - | - | - | - | - |
| 12 | 4 | 7 | 11 | 3 | 3 | 6 |
| 13 | - | - | - | 1 | 1 | 2 |
| 14 | 1 |  | 1 | - | - | - |
| 15 | 3 | 2 | 5 | 1 | - | 1 |
| 16 | - | 1 | 1 | - | - | - |
| 17 | - | 2 | 2 | - | - | - |
| 21 | - | - | - | - | - | - |
| 22 | 5 | 6 | 11 | 2 | 3 | 5 |
| 23 | 1 | - | 1 | 1 | 1 | 2 |
| 24 | - | - | - | - | - | - |
| 25 | 2 | 1 | 3 | 1 | - | 1 |
| 26 | - | 1 | 1 | - | - | - |
| 27 | - | 2 | 2 | - | - | - |
| 31 | 2 | - | 2 | - | - | - |
| 32 | 5 | 4 | 9 | 1 | 1 | 2 |
| 33 | - | - | - | - | - | - |
| 34 | - | - | - | - | - | - |
| 35 | 15 | 23 | 38 | 5 | 5 | 10 |
| 36 | 2 | 2 | 4 | - | - | - |
| 37 | - | 2 | 2 | - | - | - |
| 41 | 1 | - | 1 | - | - | - |
| 42 | 2 | 3 | 5 | - | 1 | 1 |
| 43 | - | - | - | - | - | - |
| 44 | - | - | - | - | - | - |
| 45 | 20 | 14 | 34 | 4 | 6 | 10 |
| 46 | 1 | 2 | 3 | - | - | - |
| 47 | - | 3 | 3 | - | - | - |
| Total | 64 | 75 | 139 | 19 | 21 | 40 |
| 12,22 | 2 | 6 | 8 | 2 | 2 | 4 |
| 15,25 | 2 | 1 | 3 | 1 |  | 1 |
| 16,26 | - | 1 | 1 | - | - | - |
| 17,27 | - | 2 | 2 | - | - | - |
| 31,41 | 1 | - | 1 | - | - | - |
| 32,42 | 1 | 3 | 4 |  | 1 | 1 |
| 35,45 | 11 | 12 | 23 | 2 | 3 | 5 |
| 36,46 | 1 | 2 | 3 | - | - | - |
| 37,47 | - | 2 | 2 | - | - | - |
| 15,25,35,45 | 2 | 1 | 3 | - | - | - |
| 16,26,36,46 | - | 1 | 1 | - | - | - |
| 17,27,37,47 | - | 2 | 2 | - | - | - |
| Abbreviations: FDI- World Dental Federation two-digit tooth notation (ref) | | | | | | |

**Table S2: A General Summary of Studies on the Association between Hypodontia and Dental Development**

|  | Lead Author, Year | Size of the Group with Hypodontia | Birth Year Range | Age Range of Children | Population | The applied method | Hypodontia-Dental Development |
| --- | --- | --- | --- | --- | --- | --- | --- |
| 1. | *Garn,  1961 | 172 |  |  | American | Normalized sex specific T-scores | Delay( not quantified) |
| 2. | Bailit,  1968 | 177 |  |  | Japanese |  | No effect |
| 3. | Rune,  1974 | 91 | 1944-1966 | 6-19 years | Swedish | Haavikko’s method | Delay(1.8 years for males and 2.0 years for females) |
| 4. | *Odagami,  1995 | 177 |  | 5-10 years | Japanese | Moorrees’s method | No effect |
| 5. | *Lozada,  2001 | 56 |  | 3.08-15.02 years | Columbian | Demirjian’s method | Delay(0,7 years for males and1 year for females) |
| 6. | Uslenghi,  2006 | 135 | 1975-2001 | 3.08-15.02 years | English | Haavikko’s method | Delay(1.51 years) |
| 7. | Tunc,  2011 | 70 | 1995-2003 | 5.3-12.5 years | Turkish | Tunc’s method | Delay for boys(0.3years)  No effect for girls |
| 8. | Erika,  2012 | 139 | 1989-1999 | 9-18 years | English | Haavikko’s^1^ and Demirjian’s^2^ method | Delay(1.20^1^ years and 1.64^2^ years) |
| 9. | Ben-Bassat,  2014 | 39 | 2000-2006 | 8-12 years | Israelite | Haavikko’s method+ Becker’s method | No effect |

*Not included in the forest plot for lack of necessary information.
